# Smoking heroin with cannabis versus injecting heroin: unexpected impact on treatment outcomes

**DOI:** 10.1186/s12954-019-0337-z

**Published:** 2019-12-05

**Authors:** Nirvana Morgan, William Daniels, Ugasvaree Subramaney

**Affiliations:** 1 Department of Psychiatry, School of Clinical Medicine, Faculty of Health Sciences, University of the Witwatersrand, 7 York Road, Parktown, Johannesburg, 2193 South Africa; 2 School of Physiology, Faculty of Health Sciences, University of the Witwatersrand, Johannesburg, South Africa; 3 Department of Psychiatry, School of Clinical Medicine, Faculty of Health Sciences, University of the Witwatersrand, Johannesburg, South Africa

**Keywords:** Heroin, Nyaope, Cannabis, Methods of heroin use, Treatment outcomes

## Abstract

**Background:**

In several countries, especially in Africa, the dominant method of heroin intake is smoking a joint of cannabis laced with heroin. There is no data exploring the impact of smoking heroin with cannabis on treatment outcomes.

**Aim:**

To compare treatment outcomes between people who inject heroin and people who smoke heroin with cannabis.

**Methodology:**

Three hundred heroin users were assessed on admission to inpatient rehabilitation and after treatment. We compared drug use, psychopathology, criminality, social functioning and general health between heroin injectors and heroin-cannabis smokers at treatment entry, and at 3 and 9 months after rehabilitation.

**Results:**

The sample comprised 211 (70.3%) heroin-cannabis smokers and 89 (29.7%) heroin injectors. Eighty-four percent were followed up at 3 months and 75% at 9 months. At 9 months, heroin-cannabis smokers had a higher proportion of those who relapsed to heroin use compared with intravenous (IV) users (*p* = 0.036). The median number of heroin use episodes per day was lower for IV users than heroin-cannabis smokers at both follow-up points (*p* = 0.013 and 0.0019). A higher proportion of IV users was HIV positive (*p* = 0.002). There were no significant differences in psychopathology, general health, criminality and social functioning between IV users and heroin-cannabis smokers at all three time points.

**Conclusions:**

Heroin users who do not inject drugs but use other routes of administration may have increased risk for relapse to heroin use after inpatient rehabilitation and should therefore have equal access to harm reduction treatment services. Advocating a transition from injecting to smoking heroin in an African context may pose unique challenges.

## Background

Injecting and chasing heroin are the most common methods of heroin use described in the literature. Injecting heroin is reported to pose the most harmful effects due to risks of overdose, transmission of blood borne viruses, more severe symptoms of dependence, longer heroin-using careers and higher rates of criminality and homelessness [1]. ‘Chasing the dragon’ is a method of heroin use whereby users inhale the vapour produced by heating heroin over a foil. Due to the severe harms associated with injecting, there have been harm reduction campaigns aimed at helping heroin injectors transition to inhaling heroin [2–4]. This could be a possible intervention in some African countries where there is a high prevalence of HIV and there are concerns about an increasing number of injecting heroin users. Within some contexts, however, the users’ accepted and familiar alternative to injecting heroin is smoking it in combination with cannabis.

South Africa, Kenya and Tanzania have reported a significant proportion of heroin users who smoke heroin combined with cannabis [5–8]. Data are however limited and the papers from Kenya and Tanzania are qualitative and therefore do not report on the specific numbers of heroin-cannabis smokers. In South Africa between 60 and 90% of heroin users entering treatment facilities report smoking heroin as their main method of heroin use [9]. The common street names for heroin in South Africa are *nyaope*, *whoonga* and *thai*. Typically, a user rolls a joint of cannabis, pours over a bag of nyaope and smokes it [5]. The reasons behind different routes of intake and drug combinations are not always fully understood. Unique cultural factors, pricing, availability of paraphernalia and the user’s subjective experience of the drug are said to play a role [6, 10, 11].

Different methods of drug use may however influence the severity of dependence and treatment outcomes. Furthermore, different methods of use may impact access to treatment. For example, in Kenya, access to opioid agonist maintenance treatment (OAMT) and other harm reduction services are reserved for injecting heroin users, and heroin-cannabis smokers have limited access [6]. Similarly, in South Africa, of the few state- and non-government-funded OAMT clinics, the majority exclusively serve people who inject drugs [9]. This is despite reports that roughly 23% or fewer may be injecting heroin users [12, 13].

In some African regions, much needed harm reduction services are slowly gaining impetus due to national HIV prevention and treatment campaigns for people who inject drugs. Whilst the roll-out of OAMT is much needed, it is also important to consider the larger heroin-using population and compare characteristics and treatment outcomes between heroin-cannabis smokers and heroin injectors. It is therefore worthwhile investigating different methods of heroin use and the biological, psychological and social sequelae thereof. These data may also help inform whether harm reduction campaigns that advocate a transition from injecting to smoking heroin would be advisable in areas where heroin is predominantly smoked with cannabis.

## Methodology

Participants were recruited from two state-funded drug and alcohol rehabilitation centres in the Gauteng Province of South Africa. The programmes entailed 1 week of inpatient detoxification followed by 6 to 8 weeks of psychosocial rehabilitation. Upon completion, most participants returned home and were encouraged to see their local social workers for follow-up and attend community-based self-help groups such as Narcotics Anonymous. The study was approved by the University of Witwatersrand Human Research Ethics Committee (M1704100).

A convenience sample of new admissions whose primary drug of use was heroin was screened for inclusion and exclusion criteria. In order to be enrolled in the study, participants were expected to

- have been using heroin in the months prior to admission,
- be older than 18 years of age,
- be willing to provide locator information for follow-up to occur,
- be able to provide informed consent.

Participants were assessed during rehabilitation and then followed up 3 months and 9 months after leaving inpatient rehabilitation. Participants were not compensated for their participation but were given 7 USD transport compensation if they returned to the research site for their follow-up interview. In some cases, the principal investigator (PI) did home and/or hangout-spot follow-up visits. Participants that were seen at home or hangout spots were not compensated. No telephonic interviews were done.

### Structured interviews

All baseline and follow-up interviews were conducted face-to-face by the PI who is a psychiatrist. Recruitment and data collection were conducted between July 2017 and February 2019. The PI was not part of the treating team at the rehabilitation facilities. Participants did not read or answer any of the questionnaires on their own. At baseline, a detailed socio-demographic and past substance use questionnaire created specifically for the study was administered. The Opioid Treatment Index (OTI) [14], which included sections on the past month drug use, past month injecting and sexual practices, social adjustment, past month criminal history and general health, was administered at baseline and both follow-up occasions. Drug use estimates in the OTI are based on the average use episodes of a substance per day. Drug use is expressed as a Q score which describes the frequency of drug use. A Q score of 1.00 to 1.99 indicates daily use and a score of greater than 2.00 indicates usage of more than once a day.

The Mini-International Neuropsychiatric Interview (MINI) version 7.0.2 for DSM-5 [15] was administered at baseline and both follow-ups to determine the presence of the following psychiatric conditions: major depressive episode (MDE past and current), suicidality (current, life time and future risk), manic and hypomanic episodes, social anxiety disorder (SAD), obsessive compulsive disorder (OCD), post-traumatic stress disorder (PTSD), psychotic disorders, generalised anxiety disorder (GAD) and antisocial personality disorder (ASPD). Screening for ASPD was only done at baseline interview.

HIV status was determined by participants’ self-report from prior testing. Urine was collected for a Multi-Drug Urine Test (MDUT) for as many participants who were able to provide a sample. Urine collection was unsupervised; however, a research assistant was trained to identify any unusual changes in colour, temperature or smell. The MDUT tested for the presence of opioids, cocaine, amphetamines, methamphetamine, cannabis and benzodiazepines. Those who relapsed to heroin use after rehabilitation were classified as continued heroin users (CHU). CHU was determined by two factors: self-reported heroin use and/or positive for opioids on the MDUT. Therefore, in cases where no MDUT was available, self-reported substance alone was used to determine drug use.

### Sample size estimation

Based on worst-case (for sample size) estimates of 50%, 5% precision and the 95% confidence level, a sample size of 385 would be required [16]. A sample size of 300 for this project corresponds to a precision of 5.7% (rather than 5.0%), which was considered adequate. Sample size estimation for the comparison of intravenous (IV) user and heroin-cannabis smoker groups was based on the chi-squared test (for categorical variables) with up to 4 × 2 table size, and the independent samples *t* test (for continuous variables); for the detection of a medium effect size (*w* = 0.3 and *d* = 0.5 respectively) with 80% power at the 5% level of significance, minimum sample sizes of 122 and 128 respectively are required. Sample size calculations were carried out in G*Power [17].

### Data analysis

Comparison of follow-up status (those seen and those lost to follow-up) for categorical variables was carried out by the *χ*^2^ test. Fisher’s exact test was used for 2 × 2 tables or where the requirements for the *χ*^2^ test could not be met. Continuous variables were assessed by the independent samples *t* test. Where the data did not meet the assumptions of these tests, a non-parametric alternative, the Wilcoxon rank sum test was used. Comparison of heroin-cannabis smokers and IV users was carried out analogously. Comparison of binary outcomes at enrolment, 3 months and 9 months, was performed by a generalised estimating equation (GEE) with the outcome as the dependent variable, observation point as the independent variable and participant as the repeated measure. Data analysis was carried out using SAS version 9.4 for Windows [18]. The 5% significance level was used.

## Results

### Description of sample at baseline

Over the recruitment period, 317 clients were screened. Eight did not fit the inclusion and exclusion criteria and five refused participation. A total of 304 participants signed consent and were enrolled in the study; however, four were withdrawn during baseline (BL) interviews as they were assessed as actively suicidal. The final sample thus consisted of 300 participants, 256 (85.3%) males. The median age at enrolment was 27 years (IQR 23–30 years, range 18–47 years), and 93.0% of the participants were Black/African South Africans (Table 1). Of the total, 200 (66.6%) were heroin-cannabis smokers, 89 (29.7%) were injectors and 11 (3.6%) used heroin only by chasing. Due to the small number in the chasing group and that 50% of all participants who reported chasing heroin also were heroin-cannabis smokers, they were grouped with smokers for further analyses. Fifty-nine participants (19.7%) reported smoking cannabis in the past month without heroin and of these, 37 were from the heroin-cannabis group, 22 injectors and 10 chasers. Heroin-cannabis smokers were significantly older at enrolment than IV users (27 years vs 25 years; *p* = 0.0062). The median age at first heroin use was significantly lower for IV users compared with heroin-cannabis smokers (18 vs 20 years, *p* = 0.0016). The median duration of heroin use was 7 years (IQR 4–9 year). Further details of the baseline sample have been presented in previous reports [19].

**Table 1:** Comparison between heroin-cannabis smokers and IV heroin users at baseline

Characteristic	Smokers (*n* = 211) (%)	IV users (*n* = 89) (%)	*p* value
Median age at enrolment (years) (IOR)	27 (24–30)	25 (22–28)	*0.01*
Gender			
Male	179 (84.8)	77 (86.5)	0.86
Female	32 (15.2)	12 (13.5)	
Years of schooling			
Up to 9 years	27 (12.8)	16 (18.0)	0.34
10–11 years	115 (55.4)	52 (58.4)	
Completed high school	38 (18.0)	10 (11.2)	
Employment status			
Employed (including informal employment)	114 (54.0)	48 (53.9)	> 0.99
Unemployed	95 (45.0)	40(44.9)	
Scholar	2 (0.9)	1 (1.1)	
Median age at first heroin use (years) (IQR)	20 (17–23)	18 (16–20)	*0.001*
Past month substance use			
Heroin	211 (100.0)	89 (100.0)	**-**
Cannabis	210 (95.5)	89 (55.1)	*< 0.0001*
Alcohol	38 (18.0)	11 (12.4)	0.30
Crystalmetamphetamine	35 (16.6)	23 (25.8)	0.08
Crack-cocaine	59 (28.0)	19 (21.3)	0.25
Methaqualone	38 (18.0)	17 (19.1)	0.87
Tobacco	208 (98.6	89 (100.0)	0.56
Median Q score heroin (IQR)	7.5 (4.5–10)	6 (4.5–9)	0.14
3 or more substances used (past month)	121 (57.3)	41 (46.1)	*< 0.0001*
Psychiatric comorbidities			
Major depressive episode (past)	74 (35.1)	28 (31.5)	0.59
Generalised anxiety disorder	60 (28.4)	18 (20.2)	0.15
Post-traumatic stress disorder	41 (19.4)	12 (13.5)	0.25
Any mental illness (excl ASPD*)	112 (53.1)	36 (40.4)	0.06
Median social functioning total score (IQR)	27 (23–31)	27 (22–31)	0.85
Median criminality total score (IQR)	4 (1–6)	4 (2–7)	*0.01*
Median general health total score (IQR)	20 (15–24)	20 (15–24)	0.87
HIV positive	21 (13.2)	23 (31.1)	*0.002*

*Antisocial personality disorder. *p* values in italics are significant

### Baseline substance use

At baseline, 71.3% of the participants were using two or three substances and 21.3% were using four or more substances. The most common substances used, other than heroin and cannabis, were crack-cocaine, crystalmetamphetamine and methaqualone. At baseline, there was no significant difference in the median number of past month use episodes between IV users and heroin-cannabis smokers for heroin, crystalmetamphetamine, crack-cocaine and methaqualone.

### Comparison between participants seen and lost to follow-up

Two hundred fifty-two participants (84.0%) were followed up at 3 months, and 225 (75.0%) were seen at 9 months after leaving inpatient rehabilitation. The chief reason for loss to follow-up at 3 and 9 months was unknown whereabouts of participants as families reported they were uncontactable and living on the streets (41% of participants lost to follow-up at 3 months and 46% at 9 months). Over the study period, 12 participants were not interviewed as they were incarcerated at the time of follow-up interview and four participants passed away. At 3 months, there was no significant association between those seen and those lost to follow-up in regard to demographic variables, substance history, psychopathology and HIV status. However, those followed up successfully had a higher proportion of participants living in formal housing at enrolment, compared with those lost to follow-up (*p* = 0.042). At 9 month follow-up, there were several significant differences between those seen and those lost to follow-up: those followed up successfully had a higher median duration of heroin use (7 vs 6 year; *p* = 0.033), lower proportion of those who used cannabis without heroin (*p* = 0.048), lower proportion of those with mental illness (*p* = 0.047), higher proportion of methaqualone users (*p* = 0.0098) and higher proportion of those living in formal housing (*p* < 0.0001).

### Heroin and other drug use at follow-up

At 3 months, none of the participants had received OAMT. At 9 months, five participants received intermittent OAMT prescribed by a private general practitioner. MDUTs were done on 76.6% and 88.4% of participants followed up at 3 and 9 months respectively. At 3-month follow-up, 65.1% had continued heroin use (CHU), and 72.4% had CHU at 9 months. Whilst there was no significant difference in the proportion of CHU between IV users and heroin-cannabis smokers at 3 months (*p* = 0.073), heroin-cannabis smokers had a higher proportion of CHU compared with IV users at 9 months (*p* = 0.036). The median Q score for heroin was significantly higher for heroin-cannabis smokers compared with IV users at 3 and 9 months (1.5 vs 0.3, *p* = 0.013 and 3.0 vs 1.5, *p* = 0.0019). The proportion of crystalmetamphetamine users (versus abstinence) was higher in IV users at 3 months (*p* = 0.049) and 9 months (*p* = 0.028). Of the 210 who were followed up at both 3 and 9 months, 34 (16.2%) were cannabis-only smokers (heroin abstinent) at the 3 -month follow-up. Of these, 50% went back to heroin use at 9 months.

### Pathways between heroin and cannabis use

For this analysis, we only used data from the 210 participants that were seen at all three time points. At the first follow-up, 42 (20.0%) were abstinent from cannabis and heroin. Of those who abstained from cannabis at 3-month follow-up, 29% went back to heroin (all with cannabis) at 9 months. There was no significant difference between cannabis-positive and cannabis-negative users at 3 months and heroin use at 9 months (50% vs 29%, *p* = 0.06).

### Psychopathology

At baseline, excluding ASPD, 49.3% of the participants had at least one mental illness. The most common were past major depressive episode (32.8%), generalised anxiety disorder (26.0%) and post-traumatic stress disorder (17.1%). The prevalence of mental illness remained the same at 3 months and decreased at 9 months (Table 2). At all three time points, there was no significant difference in the prevalence of mental illnesses between heroin-cannabis smokers and injectors.

**Table 2:** Treatment outcomes: comparison between heroin smokers and injectors

Characteristic	3 m f/up smokers (*n* = 180) (%)	3 m f/up IV users (*n* = 72) (%)	*p* value (3 m smokers vs IV)	9 m f/up smokers (*n* = 159) (%)	9 m f/up IV users (*n* = 66) (%)	*p* value (9 m smokers vs IV)
Past month substance use						
Heroin	124 (68.9)	41 (56.9)	0.073	123 (77.4)	40 (60.6)	*0.036*
Cannabis	145 (80.6)	39 (54.2)	*< 0.001*	142 (89.3)	43 (65.2)	*< 0.001*
Alcohol	98 (54.4)	41 (56.9)	0.78	72 (45.3)	31 (47.0)	0.88
Crystal meth	36 (20.0)	23 (31.9)	*0.05*	26 (16.4)	20 (30.3)	*0.03*
Median Q score heroin (IQR)	1.5 (0.0–3.5)	0.3 (0.0–2.5)	*0.01*	3 (0.6–5.0)	1.5 (0.0–3.5)	*0.002*
Any mental illness (excl ASPD*)	79 (43.9)	24 (33.3)	0.16	61 (38.4)	23 (34.8)	0.65
Median social functioning score (IQR)	23 (19–27)	22 (18–27	0.42	23 (19–27)	22 (18–28)	0.91
Median general health score (IQR)	11 (4–19)	11 (4.5–20)	0.78	15 (7–21)	14.5 (4–22)	0.84
Median criminality score (IQR)	0.0 (0.0–2.0)	0.0 (0.0–3.0)	0.11	0.0 (0.0–2.0)	0.0 (0.0–3.0)	0.93

*Antisocial personality disorder. *p* values in italics are significant

### Criminality, social functioning and general health

At baseline, 83.7% had engaged in crime in the past month, 32.9% at 3 months and 39.6% at 9 months. There was a significant difference in the median crime score between heroin-cannabis smokers and IV users at baseline (*p* = 0.01) but not at the 3 and 9 month follow-up points. Social functioning scores reflect data on accommodation, relationships and employment. At all time points, there were no significant differences between the median social functioning scores of heroin-cannabis smokers and injectors or in their median general health scores (Table 2).

### Injecting practices and HIV

There was a significant decrease in the proportion of IV users from 29.7% at baseline to 11.1% at 3 months (*p* < 0.0001) (Table 3). This however increased significantly to 16.9% IV users at 9 months (*p* = 0.01). Of those injecting heroin, 81% reported sharing needles at baseline, 82% and 89% at 3 and 9 months respectively. There was no significant change in the proportion sharing needles between baseline, 3 months and 9 months. At baseline and 3 months, 36% shared needles with three or more people and 43% reported the same at 9 months (Table 3).

**Table 3:** IV users and needle sharing

Characteristic	Baseline (*n* = 89) (%)	3 months (*n* = 28) (%)	*p* value (BL vs 3 m)	9 months (*n* = 38) (%)	*p* value (3 m vs 9 m)
Proportion of IV users in total sample	(89/300) 29.7	(28/252) 11.1	*< 0.0001*	(38/225) 16.9	*0.01*
No. sharing needles	72 (80.9)	23 (82.0)	0.89	34 (89.0)	0.42

Of the initial sample of 300, 47.0% did not have an HIV test in the preceding 6 months. Of those who knew their HIV status, 18.5% were HIV positive. The prevalence of HIV was higher in IV users than heroin-cannabis smokers (*p* = 0.002).

## Discussion

This is the first study to compare characteristics and treatment outcomes between heroin-cannabis smokers and injecting heroin users. It was expected that IV users would fare more poorly in all domains of treatment outcome. Contrastingly, heroin injectors demonstrated higher abstinence rates, had fewer heroin use episodes and used fewer substances compared with heroin-cannabis smokers. Heroin-cannabis smokers also did not differ significantly to injectors in regard to psychopathology, general health, social functioning and criminality. Amongst injectors however, the overwhelming majority shared needles before and after treatment. There was also a higher prevalence of HIV and crystalmetamphetamine use than amongst heroin-cannabis smokers at both post-treatment follow-up points. In analysing these results, there are a few factors to consider, namely the impact of cannabis in the overall addiction severity, the role of smoked heroin and lastly contextual factors such as local treatment services and HIV prevalence.

Interestingly, although there is a paucity of data on heroin-cannabis smokers, there are numerous animal model studies examining the synergistic interactions of the cannabinoid and opioid systems. Animal model data describe the ability of cannabinoids to prime the endogenous opioid system and attenuate the effects of opioid withdrawal [20, 21]. In this way, cannabinoids are said to readily interact with the opioid system and thereby modify behavioural responses linked to reward and relapse related phenomena. Some suggest that this potentiates the pharmaceutical benefits of cannabinoids in opioid dependence and pain management [20, 22]. It may also however provide insight into why heroin-cannabis smoking is a preferred method of use in some regions. Cross-agonism within these systems could potentially provide a ‘better’ high whilst at the same time decrease the severity of withdrawal. Further studies of heroin-cannabis smokers may provide new insights in the field of cannabinoid-opioid biochemistry.

Clinical studies assessing the impact of cannabis use in heroin users in the United States (US) and Israel found that cannabis use in people receiving OAMT did not negatively impact heroin abstinence rates [23–25]. In the US, currently there are also debates around the role of cannabis in the opioid epidemic [26]. It has been suggested that cannabis or cannabinoid products may be a less harmful alternative for those with opioid dependence and chronic pain [26, 27]. Those who refute this standpoint state that there is insufficient evidence to justify cannabis as an effective and safe analgesic and that cannabis acts as a companion drug that may increase use of opioids rather than abate it [28].

In our study, 50% of those who used cannabis only at first follow-up went back to heroin use 6 months later and 29% of those who abstained from cannabis continued heroin use later on. There was no significant difference in heroin abstention status at 9 months between those who used and did not use cannabis at the first follow-up. The data suggest that in this cohort cannabis use was not protective against heroin abstinence later on. Owing to the method of combination use, the risk for heroin relapse is expectedly higher in South Africa. There are however other challenges with relating international data to a South African cohort; from BL, a smaller proportion described using cannabis on its own (19.6%) and importantly our cohort received an abstinence-based approach and thus did not have the benefit of OAMT.

Methods of heroin use have evolved at varying time periods in different countries [29, 30]. The findings that heroin-cannabis smokers had higher rates of CHU and a greater number of daily heroin use episodes post-treatment is new. Furthermore, heroin-cannabis smokers and injectors did not differ in regard to the prevalence of psychopathology and total scores for social functioning and criminality. The similarities in these domains suggest that smoking heroin with cannabis resulted in equal levels of psychosocial distress. Cross-sectional studies in the UK describe more severe symptoms of heroin dependence in injecting users than chasers [31, 32]. A Spanish study found no major differences in the severity of heroin dependence between heroin injectors, smokers and sniffers in long-term users [33].

The median age at enrolment for IV users was lower and IV users began heroin use at a significantly younger age. This may reflect that IV users begin heroin use earlier however present sooner to rehabilitation presumably due to their concerns about the risks of injecting and sharing needles. Longer length of heroin use and higher frequency of heroin use episodes have been associated with poorer abstinence rates [34, 35]. In this study, heroin-cannabis smokers had a similar duration of heroin use and median number of heroin use episodes at baseline; therefore, it does not appear that these factors contributed to the lower abstinence rates. Additionally, some studies report poorer abstinence rates in younger patients; however, in this study, the older heroin-cannabis smoker group fared worse in regard to heroin use at 9 months [35, 36].

Research has suggested that IV use may result in higher peak serum concentrations of heroin but faster metabolism. Smoking heroin on the other hand results in direct alveolar transfer of heroin to cerebral arterial blood which could facilitate greater and more distinct central nervous system toxicity [30]. The impact of heroin-cannabis smoking on the central nervous system and the dual effect of cannabis dependence may be possible reasons why the smokers in this cohort had lower rates of abstinence and used heroin more frequently. At both follow-up periods, the proportion of crystalmetamphetamine users was higher in injectors. There is some similarity to studies in the UK and US that found higher proportions of crack-cocaine or amphetamine users amongst IV users compared with heroin chasers [1, 37].

Amongst IV users, approximately 80% or more shared needles. Whilst this is in keeping with previous cross-sectional studies of IV users in South Africa [38, 39], it is concerning that in this prospective study, there was no positive change after rehabilitation. As expected, the prevalence of HIV was higher in IV users; however, unexpectedly, general health scores showed no significant difference between IV users and heroin-cannabis smokers at treatment entry and thereafter. There is growing concern about the risks for chronic obstructive pulmonary disease (COPD), asthma and pneumonia in cannabis smokers [40]. In view of the fact that there is a high prevalence of heroin-cannabis smoking in South Africa, future studies should explore the prevalence of COPD in this population group. Psychiatric and non-psychiatric comorbidities in both heroin-cannabis smokers and injectors can be addressed by decreasing barriers to treatment, increasing access to treatment and implementing harm reduction-based interventions for heroin user in South Africa. The high rate of needle sharing, before and after detoxification, in a region with the highest HIV prevalence rate in the world reaffirms the shortcomings of abstinence-based treatment and an urgent need for needle exchange programmes and OAMT in South Africa. Additionally, continued heroin use amongst both injectors and heroin-cannabis smokers was high. Unlike many other prospective treatment outcome studies of patients receiving OAMT [34, 41, 42], heroin use in both injectors and heroin-cannabis smokers increased over time, suggesting that OAMT may improve outcomes in a South African heroin users seeking treatment.

This study whilst novel and robust in its results has some limitations. Firstly, we were only able to compare short-term results between heroin-cannabis smokers and injectors. It would be valuable to follow the cohort over a longer period to assess whether short-term trends are maintained. Secondly, the drug use section of the OTI takes into account the last three occasions of substance use in the past month. In this cohort, the majority of participants consumed heroin and cannabis daily at all three time points. Therefore, the Q score for heroin and cannabis is largely reflective of the average use episodes in the 3 days prior to interview. Day to day substance consumption may be affected by variables such as availability of money, availability of the substance and socialisation. Therefore, some may argue that assessing past three occasions limits the overall interpretation of the quantity and frequency of use. Lastly, this study did not specifically assess severity of dependence using a separate tool or scale. It rather looked at drug use, social functioning, injecting behaviour, criminality, general health and psychopathology as key treatment outcomes after inpatient rehabilitation. Whilst data is available regarding the frequency of heroin use, occasions of heroin use do not necessarily signify exposure when comparing different methods of use.

## Conclusions

Compared to IV users, heroin-cannabis smokers had a higher proportion who had relapsed to heroin use 9 months post-rehabilitation. The findings therefore suggest that heroin-cannabis smokers should have equal access to OAMT and should be included in policy development towards harm reduction in heroin users. A harm reduction approach that supports injecting users’ transition to heroin smoking may increase the number of heroin-cannabis smokers in South Africa. Whilst this may decrease certain harms associated with injecting, it may unfortunately also pose further risks for more severe dependence on both cannabis and heroin.

## Data Availability

The datasets generated and/or analysed during the current study are not publicly available due participant anonymity but are available from the corresponding author on reasonable request.
